# Forensic personal identification using two-dimensional computed tomographic imaging of the frontal sinus: a comparative analysis of feature matching algorithms

**DOI:** 10.1007/s00414-025-03564-5

**Published:** 2025-08-12

**Authors:** Suguru Torimitsu, Yukiko Uemura, Shigeki Tsuneya, Numfon Tweeatsani, Maiko Yoshida, Daisuke Yajima, Go Inokuchi, Ayumi Motomura, Fumiko Chiba, Yumi Hoshioka, Hirotaro Iwase, Yohsuke Makino

**Affiliations:** 1 Department of Forensic Medicine, Graduate School of Medicine, The University of Tokyo, Tokyo, 113-0033 Japan; 2 Education and Research Center of Legal Medicine, Graduate School of Medicine, Chiba University, Chiba, 260-8670 Japan; 3 Department of Forensic Medicine, School of Medicine, International University of Health and Welfare, 4-3 Kozunomori, Narita, 286-8686 Chiba Japan

**Keywords:** Personal identification, Antemortem computed tomography, Postmortem computed tomography, Accelerated-KAZE, ORB, Japanese

## Abstract

**Background:**

Personal identification plays a crucial role in forensic investigations. The frontal sinus (FS), due to its unique anatomical characteristics, is often used for human identification with radiographic imaging. This study evaluates the feasibility of personal identification using computed tomographic (CT) images of the FS with feature matching programs, the Accelerated-KAZE (AKAZE) and Oriented Features from Accelerated Segmented Test and Rotated Binary Robust Independent Elementary Features (ORB).

**Methods:**

CT images of 180 deceased individuals (135 males, 45 females) were analyzed. Antemortem (AM) and postmortem (PM) CT images of the FS were processed and compared using the AKAZE and ORB algorithms. The similarity between the paired AMCT and PMCT images was calculated using brute-force matching. Welch’s t-test was used to assess differences in similarity scores between the matched and mismatched groups. Receiver operating characteristic (ROC) analysis was used to evaluate the accuracy of the identification.

**Results:**

The mean similarity scores of the matched groups were significantly lower than those of the mismatched groups across all subgroups (*p* < 0.001). ROC analysis showed that AKAZE had a higher true positive rate (TPR = 0.950) and a lower false positive rate (FPR = 0.129) than ORB (TPR = 0.883, FPR = 0.172) for all samples.

**Conclusions:**

Feature-matching algorithms applied to CT images demonstrated high reliability for personal identification. AKAZE performed better than ORB in distinguishing between the matched and mismatched groups. These findings support the potential application of this method to forensic investigations, particularly where DNA, fingerprints, and dental materials are not available.

**Supplementary Information:**

The online version contains supplementary material available at 10.1007/s00414-025-03564-5.

## Introduction

Identification is a complex, systematic, and standardized procedure falling within the scope of civil and criminal law conducted to recover a victim’s identity and personal information [1]. Personal characteristics that are known to have unique aspects are evaluated for human identification. First, primary methods such as fingerprint, dental, and genetic analyses are considered [2]. However, the lack of available primary evidence justifies the evaluation of secondary sources of identification information [3]. Imaging can be an essential support for anthropology performed in the personal identification process [4]; unique anatomical skeletal features can be visualized through image analysis.

Due to its diversity and individuality in shape and size, the frontal sinus (FS) is one of the most interesting parts of the human body. In fact, even homozygous twins may have different FS because of pneumatization [3, 5–7]. Anatomically, the FS consists of cavities on both sides of the frontal bone that are only visible on radiographic images around the age of four [8–10]. The FS is fully grown by approximately the age of 19 [11, 12]. Except in circumstances of pathology, trauma, or surgery, the FS does not change markedly until old age, when pneumatization may increase due to atrophic changes [13–17]. In addition, even in damaged remains, the FS is relatively preserved due to the stability of the frontal bone [4, 18]. Thus, morphological and metrical analysis of the FS can be practically used to identify unknown corpses [19–22]; comparative identification of the FS using data from antemortem (AM) and postmortem (PM) radiographic images is a major research area in forensic medicine [8, 23–28].

Since the introduction of modern forensic imaging such as computed tomography (CT) and magnetic resonance imaging into forensic medicine in the 1990 s, the use of PMCT to supplement autopsies has continuously increased worldwide [29–33]. PMCT has become the preferred method of radiological identification of decedents and is recommended for the identification of disaster victims [6, 34–36]. Many forensic institutes routinely perform CT scans before autopsies and have a continuously growing data set of contemporary populations that may be suitable for research [27, 37]. In addition, CT is already used in the diagnosis and treatment planning of several clinical conditions. Furthermore, CT provides information regarding the pneumatization pattern of the FS even when it is not the primary area of interest in the examination of maxillofacial conditions, such as facial trauma [24, 38]. Thus, the paranasal sinuses and FS in particular are one of the most commonly analyzed anatomical features, which have been shown to be very reliable when imaged using two-dimensional (2D) conventional radiology, three-dimensional (3D) CT, and cone-beam CT [3, 4, 6, 14, 28, 39–43]. Among these, the methods based on 2D imaging were observed to be popular, probably due to their low cost and accessibility, in contrast to the remaining 3D methods [21].

Feature matching, which is an image comparison program to detect unique points that can be distinguished from others, is a new personal identification method that produces quantified similarity [44]. Feature matching for an image can be referred to as 2D object recognition [45]. However, no research has compared AMCT and PMCT images of the FS using feature descriptor algorithms such as Accelerated-KAZE (AKAZE) and Oriented Features from Accelerated Segmented Test (FAST) and Rotated Binary Robust Independent Elementary Features (BRIEF) (ORB). This study examined the feasibility of personal identification with 2D CT images of the FS using AKAZE and ORB.

## Materials and methods

### Materials

The study protocol was approved by the Ethics Committees of the University of Tokyo (10835) and Chiba University (2819). The sample comprised AMCT and PMCT scans of 180 adult Japanese corpses of known age and sex (135 males; 45 females) obtained from the Department of Forensic Medicine at the University of Tokyo and the Education and Research Center of Legal Medicine at Chiba University between December 2009 and November 2024 (AMCT) and between July 2013 and November 2024 (PMCT). The male and female samples were 19–98 (mean: 60.8 ± 18.6) years and 19–93 (mean: 63.9 ± 23.8) years of age at death, respectively; there were 81 cases aged under 60 years old, and there were 99 cases aged 60 years or older. The interval between the AMCT and PMCT scans ranged from 3 to 1920 (mean: 156.6 ± 434.7) days; there were 125 samples with an interval of less than 30 days and 55 samples with an interval of 30 days or more. Exclusion criteria were skull fractures, fatal head trauma, burn injuries, and acquired or congenital abnormalities affecting normal morphology and/or the ability to obtain reliable data.

### Methods

AMCT scanning was performed at hospitals in Japan using the CT systems listed in Supplementary Table 1 with a slice thickness of 0.63 mm to 10 mm (median, 5.0 mm), a tube voltage of 120 kV to 140 kV (median, 120 kV), and a tube current of 94 mA to 665 mA (median, 270 mA). The images were reconstructed to the same thickness as the scanning protocol.

PMCT scanning was performed using a 16-row detector CT system, either Eclos (Fujifilm Co., Tokyo, Japan) or Supria Advanced FR (Fujifilm Co., Tokyo, Japan). The scanning protocol was as follows: collimation of 0.625 mm, reconstruction interval of 0.625 mm, tube voltage of 120 kVp, and tube current of 200 mA or auto regulation.

**Fig. 1:**
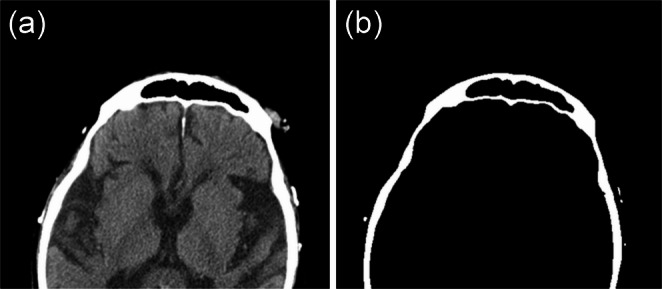
(**a**) A representative axial CT image showing the frontal sinus; (**b**) an image of (a) in which CT values less than 200 HU are displayed in black and those 200 HU and over in white

The image data, reconstructed using a soft tissue kernel, were processed on a workstation, Synapse Vincent (Fujifilm Co., Tokyo, Japan), to obtain orthogonal multiplanar reconstruction (MPR) images. For the AMCT images, a representative image of an axial section showing the FS was selected for each case. CT values of less than 200 HU were displayed in black and those of 200 HU and over in white, and the images with the shape of the FS in black and the surrounding bone area in white were extracted and saved (Fig. 1). The PMCT images were adjusted using MPR arbitrary sections, and a representative image of an axial section showing the FS, which was similar in shape to the FS in the selected AMCT image, was obtained for each case. Then, similar to the AMCT images, image processing was performed on the CT values. The saved images were processed using Adobe Photoshop^®^ (Adobe, San Jose, CA, USA) to a size of 200 × 100 pixels, with only the FS in black on a white background.

**Fig. 2:**
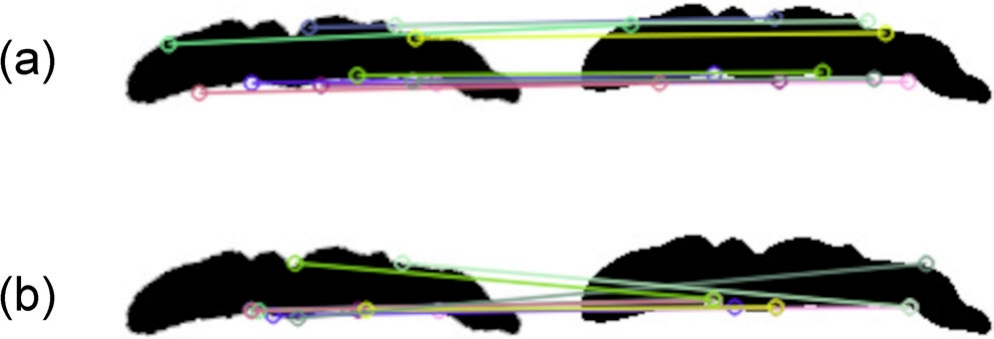
Image comparison using (**a**) Accelerated-KAZE and (**b**) Oriented Features from Accelerated Segmented Test (FAST) and Rotated Binary Robust Independent Elementary Features (BRIEF); matched feature points are drawn on the output images

AKAZE is a keypoint extraction method that was developed by Alcantarilla et al. [46] in 2013 from an algorithm called KAZE feature descriptor [47]. AKAZE uses Fast Explicit Diffusion to construct a nonlinear scale space for scale independence. Following this, keypoint extraction is performed for each image in a nonlinear scale space. Finally, a scale- and rotation-invariant Modified Local Difference Binary method is used as a descriptor of the texture to extract features at keypoints [46]. AKAZE can be used to determine whether objects that are photographed from different angles are identical, making comparative judgments by calculating the feature values of two images quantifying the similarity between images by comparing the calculated values [44]. Figure 2a shows a comparison of images developed using AKAZE, which can detect comprehensive features with high recognition accuracy, has better processing speed than classical keypoint extraction algorithms such as Scale-Invariant Feature Transformer (SIFT) [48] and Speeded-Up Robust Features (SURF) [49], and is robust to rotation and dilation [44]. In addition, AKAZE is free of patent approval, and its software implementation is relatively simple.

ORB is a high-speed binary descriptor and a robust local feature detector that was developed in OpenCV lab by Rublee et al. [50] (Fig. 2b). It uses the FAST keypoint detector, the Harris corner detector, and the BRIEF keypoint descriptor to perform feature extraction [51–53]. In SIFT; the Euclidean norm is used to provide spatial distance matching, while the Hamming distance is used for binary descriptors, such as ORB [54]. In addition, the use of ORB could considerably increase system scalability and computational efficiency, such as in the case of storage memory, time, and the central processing of unit computations. Furthermore, the Hamming distance can be used for string comparison or matching. This can be done quickly, using modern CPUs through performing XOR or bit-count operations. ORB on modern computers can be used for rapid similarity matching [54]. In fact, the ORB has been shown to provide faster and more accurate results in relation to SIFT and SURF [55]. In addition, as with AKAZE, ORB is licensed for free, unlike the patented SIFT and SURF algorithms [52].

The similarity scores produced by AKAZE and ORB were defined as the average Hamming distance of the feature values between the two images. The lower the similarity score, the more similar the images, such that similarity is 0 when the images are exactly the same.

Image-comparison programs based on the AKAZE and ORB were implemented and used with the OPENCV library in Python [44, 45]. The AMCT and PMCT images for all 180 samples were compared using brute-force matching. Then, brute-force matchings of AMCT and PMCT images were performed for each of the following subgroups: male, female, CT scanning interval of less than 30 days, CT scanning interval of 30 days or more, aged less than 60 years, and aged 60 years or older. The similarity scores obtained were tested for mean differences between the matched (identical-sample) and mismatched (different-samples) groups using the Welch test (p values less than 0.05 were considered to indicate significance). The comparative analysis was presented in terms of the parameters of area under the curve, cutoff values, true positive rate (TPR), and false positive rate (FPR) using receiver operating characteristic (ROC) analysis.

The first author (ST) recollected and processed the PMCT images of a randomly selected subset of 20 subjects in two different sessions separated by a 1-month interval to assess intraobserver errors; a coauthor (YU) collected the subset PMCT images to assess interobserver errors. Intra- and interobserver errors were statistically quantified using the relative technical error of measurement (rTEM, %) and the coefficient of reliability (R). The acceptable ranges of rTEM for intra- and interobserver errors were < 1.5% and < 2.0%, respectively [56]. Values of R, which indicates the proportion of the between-subject variance free of measurement errors, > 0.75 were considered to indicate sufficient precision [57, 58].

The statistical analyses were performed using Excel (Microsoft Office 2019, Microsoft, Redmond, Washington, USA) and JMP ^®^ Pro 18.1.1 (SAS Institute Inc., Cary, NC, USA).

## Results

The raw study data for age, sex, CT scan interval, and similarity scores between the AMCT and PMCT images are presented in Supplementary Material 2. The similarity scores used to evaluate intra- and interobserver errors are shown in Supplementary Material 3. Table 1 shows the rTEMs and R values; the rTEMs for intraobserver and interobserver errors were < 1.5% (1.39–1.43%) and < 2% (1.77–1.88%), respectively; the R values were > 0.75 (0.95–0.99).

**Table 1:** The relative technical error of measurement (rTEM) and the coefficient of reliability (*R*) (*n* = 20)

Method	Intra-observer			Inter-observer	
	rTEM	*R*		rTEM	*R*
AKAZE^a^	1.43%	0.99		1.77%	0.98
ORB^b^	1.39%	0.97		1.88%	0.95

^a^ Accelerated-KAZE

^b^ Oriented FAST and Rotated BRIEF

The descriptive statistics for the matched and mismatched groups using AKAZE and ORB are presented in Tables 2 and 3. The mean similarity scores for the matched group were significantly smaller than those for the mismatched group, both with respect to the total sample and for all subgroups (*p* < 0.001).

**Table 2:** Descriptive statistics of similarity scores and results of comparison using welch’s *t*-tests for similarities of AKAZE

Sample	Number	Matched			Mismatched		*P* value
		Range	Mean ± SD^a^		Range	Mean ± SD	
All	180	39.07–76.91	53.88 ± 7.06		39.07–137.78	79.54 ± 12.86	< 0.001*
Sex							
Male	135	39.62–76.91	54.34 ± 7.20		42.65–137.78	79.63 ± 13.69	< 0.001*
Female	45	39.07–67.69	52.49 ± 6.51		48.51–119.16	79.58 ± 9.84	< 0.001*
Interval							
Less than 30 days	125	39.07–76.91	55.04 ± 7.19		44.78–134.10	81.10 ± 11.75	< 0.001*
30 days or more	55	40.62–64.77	51.24 ± 6.04		44.51–123.18	76.59 ± 13.98	< 0.001*
Age							
Less than 60 years old	81	39.62–76.91	53.95 ± 8.05		43.08–137.78	79.39 ± 14.32	< 0.001*
60 years old or older	99	39.07–67.69	53.82 ± 6.18		45.38–134.12	80.06 ± 11.35	< 0.001*

^a^ Standard deviation

*Significant

**Table 3:** Descriptive statistics of similarity scores and results of comparison using welch’s *t*-tests for similarities of ORB

Sample	Number	Matched			Mismatched		*P* value
		Range	Mean ± SD^a^		Range	Mean ± SD	
All	180	29.97–52.46	39.98 ± 3.88		31.04–76.60	48.63 ± 5.08	< 0.001*
Sex							
Male	135	30.38–52.46	40.27 ± 3.70		32.91–74.99	48.81 ± 5.08	< 0.001*
Female	45	29.97–47.09	39.09 ± 4.31		33.92–74.01	48.15 ± 5.13	< 0.001*
Interval							
Less than 30 days	125	29.97–52.46	39.96 ± 4.12		31.04–76.60	48.47 ± 4.86	< 0.001*
30 days or more	55	32.14–47.69	40.00 ± 3.30		33.89–68.91	48.75 ± 5.33	< 0.001*
Age							
Less than 60 years old	81	30.38–52.46	40.40 ± 3.88		32.91–74.99	49.08 ± 5.56	< 0.001*
60 years old or older	99	29.97–51.87	39.63 ± 3.87		33.42–74.80	48.34 ± 4.70	< 0.001*

^a^ Standard deviation

*Significant

The results of the ROC analysis derived using similarity scores are shown in Table 4. For the total samples, AKAZE yielded a TPR of 0.950 and an FPR of 0.129, which were superior values to the ORB method (TPR of 0.883 and FPR of 0.172). In the subgroups by sex, both TPR and FPR were slightly greater for males than for females. In subgroups according to the CT scanning interval, the TPR and FPR were both slightly larger in the group of 30 days or more than in the group of less than 30 days. In subgroups by age, according to the analysis using AKAZE, while the TPR was slightly larger in the group aged 60 years and older than in the group aged less than 60 years, the FPR was smaller in the group aged 60 years and older.

**Table 4:** Receiver operating characteristic analyses between identical and different groups using similarities of AKAZE and ORB

Sample	AKAZE					ORB			
	AUC^a^	Cutoff value	TPR^b^	FPR^c^		AUC	Cutoff value	TPR	FPR
All	0.959	65.25	0.950	0.129		0.923	44.08	0.883	0.172
Sex									
Male	0.950	65.25	0.948	0.139		0.924	44.08	0.882	0.161
Female	0.988	61.07	0.933	0.037		0.919	42.99	0.844	0.144
Interval									
Less than 30 days	0.975	67.69	0.976	0.110		0.918	44.08	0.872	0.170
30 days or more	0.950	64.77	1.000	0.211		0.927	43.97	0.909	0.181
Age									
Less than 60 years old	0.937	67.29	0.963	0.203		0.912	43.77	0.852	0.155
60 years old or more	0.984	64.77	0.970	0.071		0.933	44.38	0.919	0.192

^a^ Area under the curve

^b^ True positive rate

^c^ False positive rate

## Discussion

This study showed significant differences in the mean similarity scores of 2D CT images of FS between the match and mismatch groups and high TPR values obtained using ROC analyses. These results suggest the value of the FS with 2D computer-assisted superposition for identification screening when DNA, fingerprints, and dental materials are not available. However, in this study, the FPR values were slightly higher. If the FS on the 2D CT image has a simple shape, for example, when the FS is close to a rectangle, it is possible that the characteristic keypoints could not be extracted and the similarity scores may be low (evaluated as similar), even for the FS in different cases. The methods in this study may, therefore, be more effective when they are used in combination with other methods of personal identification or characteristic imaging findings.

In this study, the intra- and interobserver errors were small and probably negligible. The personal identification methods based on the 2D CT images of the FS using AKAZE and ORB are therefore reliable and reproducible.

The FS is very anatomically diverse with respect to size and shape, and it can be easily visualized using medical imaging modalities including X-rays and CT [59]. These features make the FS an ideal structure for the personal identification of human remains where AM records can be compared [60, 61]. The general advantages of methods that are based on radiological evaluation of the FS include the feasibility of noninvasive analyses (due to the inherent image settings) and the possibility of reanalyzing cases that are due to the digital storage of medical files [21].

In particular, CT is an excellent modality for the evaluation of the nasal cavity and the paranasal sinuses, as it allows accurate assessment not only of the sinuses but also of the degree of pneumatization and the craniofacial bones [62]. The increasing application of CT in identification protocols is primarily due to the gradual increase in the use of CT in clinical medicine and in the overall accessibility of CT imaging [63–67]. In fact, the widespread availability of CT in developed countries has led to a major shift from skull radiographs to head CT in diagnostic evaluations of head trauma, headache, and sinus disease in both adults and children [66–68].

Previous studies have been conducted to identify individuals using CT images of FS, and it has been reported that the results could be used in forensic investigations [5, 20, 22, 43, 69–72]. Most previous studies of personal identification based on FS have used metric and coding methods [5, 20, 22, 70, 72]. While metric analyses are objective, specialized skill is required of the examiners, and the reliability of the measurement should be established [21, 71]. The coding methods also provide greater objectivity to the previously subjective visual assessment methods of the FS [73]. However, the metric and coding methods provide only a means of performing quick searches and narrowing down suspect remains to eliminate discrepancies [74]. In addition, Besana and Rogers [15] reported that metric analyses of the FS are more limited and should not be used for personal identification.

In evaluating the FS, the use of 3D digital models based on CT is common in the forensic and anthropological literature [20, 22, 44, 70, 72, 75–77]. However, the creation of 3D CT models may not be feasible for physicians who do not have enough time or resources [73]. However, 2D CT images have the advantage that they are less costly, are familiar to most experts, and usually have a short learning curve [21].

A large literature exists on the use of morphological and measurement features for the side-by-side comparisons based on one or more 2D FS images that are obtained from X-ray or CT scans [59, 60, 71, 78–81]. However, slight changes in the head position and different imaging methods could alter the resulting images, in particular where there are few fixed cross-sectional images, which makes the comparison process more difficult and the identification results less reliable [70]. In addition, previous studies have reported that qualitative analysis relies in part on the skill and experience of the examiner and could involve more subjectivity and intuition [3, 21, 82]. Our study objectively assessed the anatomical specificity of the FS, using the image comparison programs, providing further evidence concerning the reliability of forensic personal identification without the influence of image position or rotation.

Of the programs used in this study, AKAZE produced better results than ORB. Alcantarilla et al. [47] reported that the AKAZE algorithm is more efficient than previous keypoint-extraction methods, including ORB. In addition, Kubo et al. [45] presented statistically significant differences in reference to the same and different models in dental information images with AKAZE. These results indicate the usefulness of AKAZE for human identification. Kubo et al. [45] reported that it took approximately 3 to 5 min to create a cross-sectional image and 1 min to compare images if AKAZE had been implemented. Previous studies have found that the nasal septum and paranasal sinuses other than the FS, such as the sphenoid sinus, are useful for personal identification [28, 83–85]. Additional studies should be conducted to identify individuals using AKAZE, based on the peri-FS sites.

Previous studies reported that males have larger muscles and organs than females, and their lungs, nasal cavities, and sinuses are also correspondingly larger, which indicates the importance of sinus evaluation as a method of forensic identification and sex determination using CT [62, 77, 86–88]. However, there were no apparent sex differences in the TPR or FPR for personal identification in this study, which indicates that personal identification using the image comparison programs was not significantly affected by sex differences. Note that the sample size of females in this study was smaller than that of males; further studies with larger sample sizes are thus warranted.

In the present study, few differences were found in either TPR or FPR between the subgroups distinguished by CT scan interval. Kirk et al. [13] reported that the ability of FS to identify a corpse by visual inspection is not affected by the time that has elapsed between the AM and PM radiographs. This suggests that AMCT and PMCT images could be used for personal identification even when there is a long interval between images acquisition. However, because many of the samples in this study had short intervals between the AMCT and PMCT images, further research should use more samples with long intervals between the AMCT and PMCT images taken to test this finding.

Tatlisumak et al. [89] reported that the lowest FS measurements were obtained after age 60. However, there was no objective explanation for this point. On the contrary, some studies [90–92] have found that the FS may expand in old age due to resorption of the bone walls, which could affect reliable identification of the remains. In this study, few differences were found in terms of TPR and FPR between the subgroups by age. These results suggest that 2D CT images of FS could be used for personal identification, not only in the young but also in the elderly.

There are several limitations to this study. First, the CT images employed were obtained using various equipment and imaging conditions. The reliability of the results of the superimposition is closely related to the 2D segmentation and reconstruction of the FS in terms of whether the FS could be ideally depicted based on the CT threshold values; thus, the information of the image itself is important [70]. Previous studies have reported that different procedures of data acquisition, such as different scanning protocols, CT equipment, and orientations between AMCT and PMCT images, may affect the segmentation accuracy of the same subject [70, 81, 93, 94]. Therefore, it is desirable to use the same CT equipment and conditions for both AMCT and PMCT images. However, it is practically difficult because the subjects of AMCT and PMCT images are different, being the patients and the corpses.

Second, the method used in this study is only applicable if AMCT images of the person being compared are available for comparison with the PMCT images obtained from the corpse [4, 21].

Third, the method employed in this study was not fully automated, and it did not automatically generate FS images from CT scans of the head, which required manual intervention in the dataset. While the intra- and interobserver errors were small, the efficiency of the method was certainly reduced. However, Souza et al. [69] have noted that, although CT images do indeed have high contrast, fully automated segmentation is necessarily problematic due to the complexities of image processing.

Fourth, although no samples in this study had bilateral FS aplasia, bilateral sinus aplasia was observed in 4–10% of the samples, depending on the sample origin [73, 95]. When comparing individuals with sinus aplasia, the methods applied in this study may not be available, and alternative methods should be considered for personal identification. In addition, population should be considered when assessing the FS [77]; studies similar to this present study in other population groups should be conducted.

Finally, the size of the FS may be related to genetic factors [40]. Stress, masticatory function, and changes in growth hormones may also affect FS morphology [96]. In addition, other conditions, such as trauma, surgery, and frontal sinusitis can cause changes in the morphology of the FS [61, 70]. However, in this study, cases with trauma to the sinuses were excluded. Although all of these factors should be considered, the information available to the forensic investigators is limited. Furthermore, it should be noted that metallic objects close to the sinus cavity, such as dental implants and foreign objects, can cause artifacts that lead to the distortion of the surrounding anatomical structures [43, 97].

## Conclusions

This study showed that forensic personal identification using 2D CT images of the FS is a reliable and reproducible method when applying feature descriptor algorithms. AKAZE outperformed ORB in accuracy, with higher TPR and lower FPR values. These findings suggest that FS image comparison can be a valuable tool for forensic investigations, in particular when primary identification methods such as DNA, fingerprint, and dental analyses are not available. However, further research is needed to refine the automated segmentation techniques, explore population-based variations, and assess the use of this method in diverse forensic scenarios.

## Electronic supplementary material

Below is the link to the electronic supplementary material.

Supplementary Material 1

Supplementary Material 2

Supplementary Material 3

## Data Availability

The datasets generated during and/or analysed during the current study are available from the corresponding author on reasonable request.
